# Genome Sequence of *Hydrogenovibrio* sp. Strain SC-1, a Chemolithoautotrophic Sulfur and Iron Oxidizer

**DOI:** 10.1128/genomeA.01581-17

**Published:** 2018-02-01

**Authors:** Christopher Neely, Charbel Bou Khalil, Alex Cervantes, Raquel Diaz, Angelica Escobar, Karen Ho, Stephen Hoefler, Hillary H. Smith, Karla Abuyen, Pratixaben Savalia, Kenneth H. Nealson, David Emerson, Benjamin Tully, Roman A. Barco, Jan Amend

**Affiliations:** a Department of Earth Sciences, University of Southern California, Los Angeles, California, USA; b Department of Biological Sciences, University of Southern California, Los Angeles, California, USA; c Department of Environmental Studies, University of Southern California, Los Angeles, California, USA; d Center for Dark Energy Biosphere Investigations, University of Southern California, Los Angeles, California, USA; e Bigelow Laboratory for Ocean Sciences, East Boothbay, Maine, USA; f Rio Hondo Community College, Whittier, California, USA; g Pasadena City College, Pasadena, California, USA; h Brookdale Community College, Middletown, New Jersey, USA; i Mission College, Santa Clara, California, USA; j Orange Coast College, Costa Mesa, California, USA; k Santiago Canyon College, Orange, California, USA; l Waubonsee Community College, Sugar Grove, Illinois, USA; m Community College Cultivation Cohort, University of Southern California, Los Angeles, California, USA

## Abstract

*Hydrogenovibrio* sp. strain SC-1 was isolated from pyrrhotite incubated *in situ* in the marine surface sediment of Catalina Island, CA. Strain SC-1 has demonstrated autotrophic growth through the oxidation of thiosulfate and iron. Here, we present the 2.45-Mb genome sequence of SC-1, which contains 2,262 protein-coding genes.

## GENOME ANNOUNCEMENT

Iron is an essential trace element that limits primary productivity in surface marine waters (1). Microaerophilic neutrophilic iron-oxidizing bacteria are involved in the cycling of iron in the marine environment; however, this biological function has so far been predominantly associated with representatives of the class *Zetaproteobacteria* (2) and with other uncharacterized isolates (3). Here, we present the genome sequence of *Hydrogenovibrio* sp. strain SC-1, which was isolated from pyrrhotite coupons that were incubated *in situ* on surface marine sediments of Big Fisherman’s Cove, Catalina Island, CA. SC-1 is the only known iron-oxidizing bacterium within the family *Piscirickettsiaceae* (4). Similar to the iron-oxidizing *Zetaproteobacteria*, SC-1 is able to use the energy from iron to grow autotrophically (5). Strain SC-1 is a member of the *Thiomicrospira-Hydrogenovibrio-Thiomicrorhabdus* group of bacteria, which is known for autotrophic thiosulfate oxidation and obligate chemolithoautotrophy (6).

SC-1 genomic DNA was extracted by bead beating using the FastDNA Spin soil kit (MP Biomedical, Santa Ana, CA, USA) per the manufacturer’s protocol. DNA was sequenced at the Single Cell Genomics Center at the Bigelow Laboratory for Ocean Sciences using a NextSeq instrument (Illumina, USA). A total of 6,336,875 raw paired-end sequences were generated and processed using Trimmomatic version 0.32 (7) to trim the last 5 bp of each sequence, regions with low quality scores (*Q* < 15), and reads less than 36 bp in length, resulting in 5,349,014 quality-controlled sequences. As part of the standard operating procedure for the Single Cell Genomics Center, quality-controlled sequences were processed prior to assembly using a complexity filter threshold of 0.05, normalization with kmernorm version 1.05 (parameters: *k* = 21; *t* = 30; *c* = 3) (http://sourceforge.net/projects/kmernorm/), and a contamination filter with an identity threshold of 1, yielding 2,313,229 high-quality paired-end sequences (8). These sequences were *de novo* assembled using the SPAdes genome assembler version 3.0.0 (9), generating 41 contigs. The maximum contig length was 263,867 bp. The *Hydrogenovibrio* sp. SC-1 genome was 2.45 Mb in length, with a GC content of 42.9% and an *N*_50_ value of 131,404 bp. Annotation was performed by the Joint Genome Institute (JGI) Integrated Microbial Genomes system (10), resulting in 2,262 protein-coding genes, 2 rRNAs (1 copy each of the 16S and 23S rRNA genes), and 36 tRNA genes.

Analysis revealed genes that encode proteins for the tricarboxylic acid cycle, pentose phosphate cycle, glycolysis, and carbon fixation via the Calvin cycle and for the oxidations of thiosulfate (*soxABCXYZ*) and sulfide (sulfide-quinone reductase). Genes coding for flagellum biosynthesis and chemotaxis indicate that SC-1 bacteria have the potential for motility, but this has not been observed under culture conditions. Genes that show the potential for the oxidation of molecular hydrogen (*hydAB* and* hypABCDEF*) were detected.

### Accession number(s).

This whole-genome shotgun project has been deposited in DDBJ/ENA/GenBank under the accession no. PKGB00000000. The version associated with this submission is version PKGB01000000. Also, the SC-1 genome sequence described in this paper has been deposited in the JGI Integrated Microbial Genomes and Microbiomes system and in the JGI Genome Portal under IMG Genome ID 2627853948.
